# A Molecular Model of Blood Cell Renewal

**DOI:** 10.1371/journal.pbio.0020349

**Published:** 2004-09-28

A developing organism captured on time-lapse video is a wonder to behold. If you're watching a chick embryo, by day 3, you'll see millions of cells engaged in a frenzy of activity, as rapidly dividing cells migrate to new positions, acquire the characteristics of specialized cells, and craft well-defined tissues, organs, and limbs in just under two weeks. In addition to the cells destined for specialization is another important group, stem cells, whose progeny have two very different fates. They can either “self renew”—that is, make identical copies of themselves—or generate intermediate progenitor cells that give rise to mature, differentiated cells.

Both differentiation and self renewal are guided by an elaborately regulated genetic program, which transforms embryonic stem cells into the many different cell types that make up the body. Adult stem cells share the hallmark trait of self renewal, but are relatively rare: in bone marrow, the source of hematopoietic, or blood-forming, only an estimated one in 10,000–15,000 cells is an adult hematopoietic stem cell (HSC).

Studies that have compared the gene expression profiles of different types of stem cells to identify genetic signatures of “stemness” have found only a limited number of signature genes. And the molecular mechanisms that regulate this so-called potency and the self renewal process have remained obscure. Now, focusing on HSCs, Margaret Goodell and colleagues have undertaken a systematic evaluation of HSC renewal. The study identifies molecular signatures associated with discrete stages of the HSC self renewal cycle and proposes a molecular model of the process.

HSC renewal passes through three stages: quiescence, activation and proliferation, and a return to the dormant state. HSCs give rise to both red blood cells, which carry oxygen and carbon dioxide, and white blood cells, which fight infection. Certain stressors—including blood-cell-inhibiting chemotherapy and bone marrow transplants—trigger HSC activation, which induces rapid proliferation, generating both progenitors to deal with the threat and new stem cells that return to quiescence. Once activated by a trigger, dormant HSCs engage a regulatory program that rapidly churns out billions of cells, then puts the brakes on cell division, prompting the return to a nondividing, quiescent state.

To understand the genetic programs underlying this process, Goodell and colleagues induced proliferation in HSCs (with the chemotherapeutic drug, 5-fluorouracil, or 5FU), then allowed the cells to return to quiescence, so they could characterize the changes in gene expression that occurred during each stage. They compared these time-specific patterns to the gene expression profiles of naturally proliferating fetal mouse HSCs (which undergo massive proliferation) and quiescent adult mouse HSCs (which hardly divide at all) to find genes associated with the two different states.

Genes were grouped into proliferating or quiescent groups based on when they were expressed after 5FU treatment, and these groupings were refined based on comparisons to previously published HSC gene expression data. Functional analysis of these genes found a bias toward genes involved in cell division processes in the proliferation stage and toward cell division inhibitors in the quiescent stage, supporting the logic of the groupings. To understand the activation process at a global level, the authors employed some novel analysis strategies, including the “Gene Ontology” (GO) system for classifying genes.

With these results, Goodell and colleagues constructed a model of the HSC self renewal cycle: quiescent HSCs maintain a “state of readiness,” molecularly speaking, that allows a quick response to environmental triggers. A stressor (like the chemotherapy mentioned above) triggers a “prepare to proliferate” state—a kind of pregnant pause—and then the proliferation machinery kicks in, going through an early and late phase before quiescence returns. By shedding light on the molecular mechanisms of stem cell renewal, this study will aid efforts to develop stem-cell-based clinical therapies, which depend on replicating the HSC self renewal cycle to replenish diseased or damaged tissue, and will ultimately guide efforts to grow stem cell colonies outside the body, a long-standing goal that would have many clinical applications. The authors suggest their findings may also be relevant to studies of cancer stem cells, tumor cells with self renewal properties.

